# Synergistic effect of mesenchymal stem cell-derived extracellular vesicle and miR-137 alleviates autism-like behaviors by modulating the NF-κB pathway

**DOI:** 10.1186/s12967-024-05257-w

**Published:** 2024-05-13

**Authors:** Qian Qin, Zhiyan Shan, Lei Xing, Yutong Jiang, Mengyue Li, Linlin Fan, Xin Zeng, Xinrui Ma, Danyang Zheng, Han Wang, Hui Wang, Hao Liu, Shengjun Liang, Lijie Wu, Shuang Liang

**Affiliations:** 1 Department of Children’s and Adolescent Health, Public Health College, Harbin Medical University, Harbin, 150081 China; 2 Department of Histology and Embryology, Harbin Medical University, Harbin, 150081 China

**Keywords:** Autism spectrum disorder, Extracellular vesicles, Mesenchymal stem cell, TLR4/NF-κB pathway, miR-137

## Abstract

Autism spectrum disorder (ASD) is a multifaceted neurodevelopmental disorder predominant in childhood. Despite existing treatments, the benefits are still limited. This study explored the effectiveness of mesenchymal stem cell-derived extracellular vesicles (MSC-EVs) loaded with miR-137 in enhancing autism-like behaviors and mitigating neuroinflammation. Utilizing BTBR mice as an autism model, the study demonstrated that intranasal administration of MSC-miR137-EVs ameliorates autism-like behaviors and inhibits pro-inflammatory factors via the TLR4/NF-κB pathway. In vitro evaluation of LPS-activated BV2 cells revealed that MSC-miR137-EVs target the TLR4/NF-κB pathway through miR-137 inhibits proinflammatory M1 microglia. Moreover, bioinformatics analysis identified that MSC-EVs are rich in miR-146a-5p, which targets the TRAF6/NF-κB signaling pathway. In summary, the findings suggest that the integration of MSC-EVs with miR-137 may be a promising therapeutic strategy for ASD, which is worthy of clinical adoption.

**Supplementary Information:**

The online version contains supplementary material available at 10.1186/s12967-024-05257-w.

## Introduction

Autism spectrum disorder (ASD) encompasses a series of complex neurodevelopmental disorders, mainly manifested before the age of three. These disorders are characterized by diverse levels of impairment in social interaction and communication as well as restricted or repetitive behaviors and interests [1]. Epidemiological surveys show that the incidence of ASD is increasing globally, annually, and exponentially. 2023 data from the United States Centers for Disease Control and Prevention showed that 2.76% of 8-year-old children had been diagnosed with ASD [2]. ASD requires specialized educational, healthcare, and familial support services, which bring enormous economic and emotional pressure to societiy and family [3]. Therefore, ASD has escalated into a major public health issue, seriously affecting the quality of life and overall population health, making the exploration of its etiology and treatment essential crucial in contemporary medical research.

Extracellular vesicles (EVs) encapsulate distinctive bioactive molecules reflecting the composition and physiological statuses of eukaryotic cells. These vesicles promote various modes of cellular target engagement, and facilitate intercellular information transmission [4–6]. Studies have shown that exosomes can play pivotal roles in the therapeutic intervention of nervous system disorders [7]. In addition, the lipid bilayer structure of EVs protects their contents from degradation, enhancing their important role in intercellular communication [8].

Mesenchymal stem cells (MSCs) are pluripotent stem cells with various differentiation potential, immune regulation ability, tissue repair ability, and neuroprotective ability, among other functionalities. In recent years, they have become increasingly prominent and widely used in the treatment and research of multisystem disorders such as neurological and cardiovascular diseases [9]. MSCs are known to alleviate neuroinflammation, protect neurons, promote neuronal axon growth, and enhance neuroregeneration. Compared to MSCs, MSC-derived extracellular vesicles (MSC-EVs) present a more stable option, with stronger preservation ability and a reduced risk of immune rejection, thus providing innovative pathways for cell therapy of various diseases. Research has confirmed that MSC-EVs can reduce pro-inflammatory agents such as interferon-γ (IFN-γ) and tumor necrosis factor (TNF-α), thereby attenuating the inflammatory response [10].

MicroRNAs (miRNAs) are small regulatory RNAs prevalent in various organisms and are instrumental in modulating post-transcriptional gene expression by directing mRNA toward degradation or inhibiting translation [11]. As a specific example, miR-137 modulates neuronal synaptic length and influences neuronal maturation and dendritic morphogenesis, which are pivotal in determining neuronal structure and functionality [12, 13]. Early studies have discerned associations between miR-137 dysfunction and various neuronal aspects such as differentiation, inflammation, and overall neurodevelopment, as well as the emergence of neurological disorders such as schizophrenia [14–16]. Notably, the Institute of Animal Science, Chinese Academy of Sciences has reported that mice with a nervous system-specific knockout of miR-137 exhibit phenotypic irregularities, such as stereotypical repetitive behaviors and deficits in social competency and learning memory [17]. These findings underscore the potential significance of miR-137 in the genesis and progression of ASD, necessitating further in-depth investigation and elucidation.

This study aims to explore the potential mechanisms of MSC-EVs and MSC-miR137-EVs in alleviating autism-like behaviors. These findings can offer novel perspective on targeted intervention for ASD.

## Results

### Preparation and characterization of MSC-EVs and MSC-miR137-EVs

**Fig. 1:**
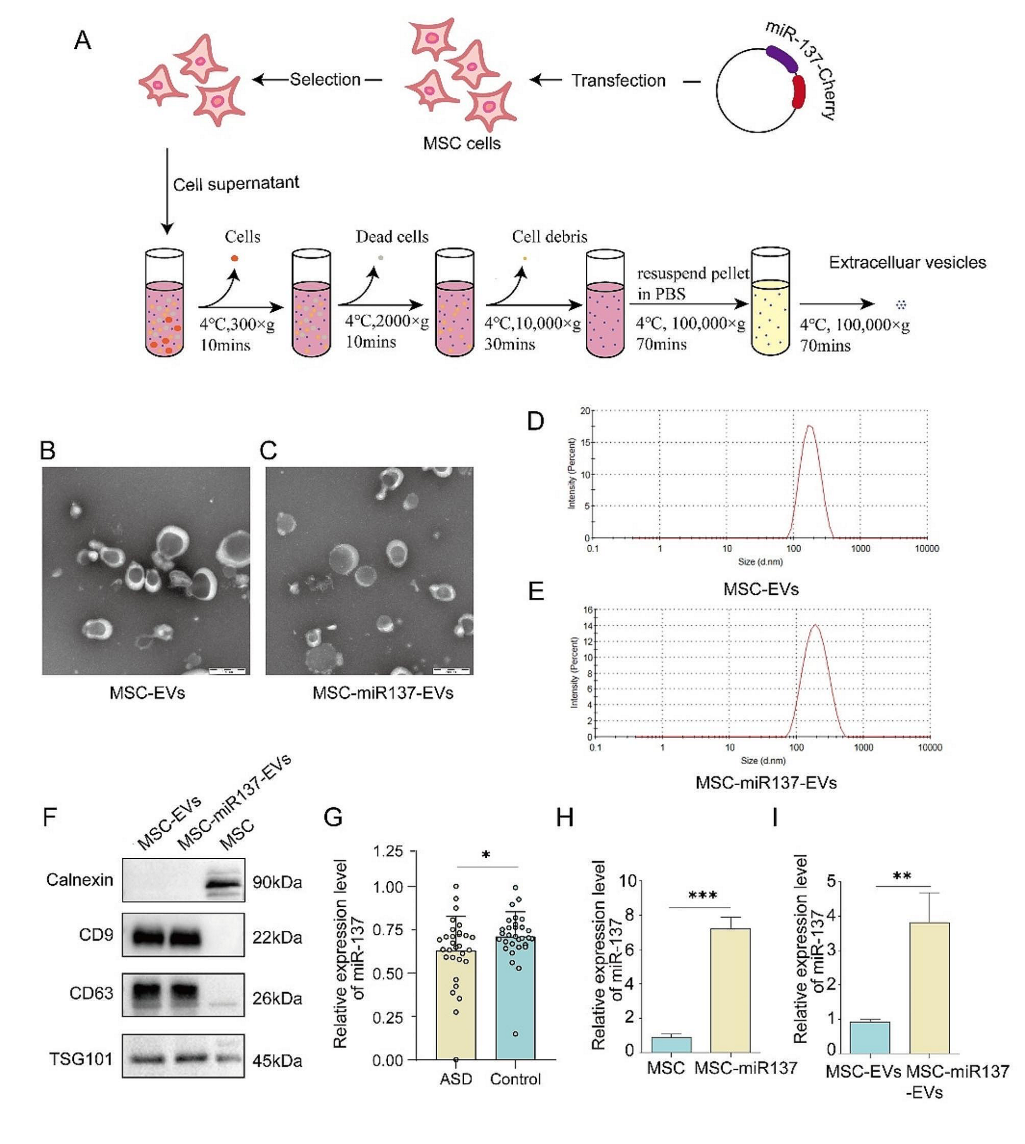
Preparation and characterization of MSC-EVs and MSC-miR137-EVs. **(A)** Schematic illustrating the production and harvest process of MSC-miR137-EVs for targeted miR-137 delivery. **(B–C)** TEM images of EVs isolated from the culture medium of MSCs. **(D–E)** Size distribution of MSC-EVs and MSC-miR137-EVs measured using a nanoparticle size meter. **(F)** Western blot analysis demonstrating the expression of CD9, CD63, TSG101, and calnexin in MSC-EVs and MSC-miR137-EVs. **(G)** The miR-137 expression level of peripheral blood between ASD patients and controls. **(H)** Relative qRT-PCR analysis of miR-137 in MSCs-miR137 transduced with miR-137 lentivirus. ***P < 0.001 versus the MSC group using Student’s *t* test. **(I)** Relative qRT-PCR analysis of miR-137 in MSC-miR137-EVs. **P < 0.01 versus MSC-EVs using Student’s *t* test. All data are presented as the mean ± SEM of three independent experiments

To construct MSC-miR137-EVs, lentiviruses overexpressing miR137 were transduced into MSC cells. MSC-EVs and MSC-miR137-EVs were then isolated from MSCs and MSCs expressing miR-137 (MSCs-miR137), respectively, using gradient centrifugation and ultracentrifugation method (Fig. 1A). The isolated EVs exhibited physical homogeneity when observed under transmission electron microscopy (TEM) (Fig. 1B, C). Meanwhile, nanoparticle analysis revealed an average size of 180 nm in diameter, as illustrated in Fig. 1D, E. Additionally, the presence of characteristic EVs membrane proteins such as CD9, CD63, and tumor susceptibility gene 101 (TSG101), along with the intracellular protein calnexin, was verified through Western blot analysis, further substantiating the identification of the isolated particles as EVs (Fig. 1F). In this study, the gene expression profile within the GSE89596 dataset, which is focusing specifically on miRNA levels of peripheral blood in ASD patients with control of the public Gene Expression Omnibus (GEO) database was download and analyzed. Our analysis confirmed that miR-137 expression was significantly lower in ASD patients than that in control (Fig. 1G). Subsequently, quantitative real-time polymerase chain reaction (qRT-PCR) was employed to assess the levels of miR-137 in MSCs transfected with the lentiviral expression vector. As shown in Fig. 1H, the expression of miR-137 was elevated in MSCs-miR137 compared to that in MSCs. Furthermore, the expression of miR-137 in MSC-miR137-EVs was higher than that in MSC-EVs (Fig. 1I). These results collectively indicated the successful construction of MSC-EVs and MSC-miR137-EVs, confirming their suitability for subsequent studies.

### Engineered MSC-EVs efficiently delivered exosomal miR-137 into the brain

The BTBR T + Itpr3tf/J (BTBR) mouse model emulates numerous core clinical manifestations typically observed in patients with ASD, such as comparable behavioral attributes, brain structures, and functional abnormalities [18]. This resemblance makes the BTBR mouse model exceptionally suited for ASD research. Consequently, BTBR mice were selected for investigating the in vivo role of MSC-miR137-EVs.

**Fig. 2:**
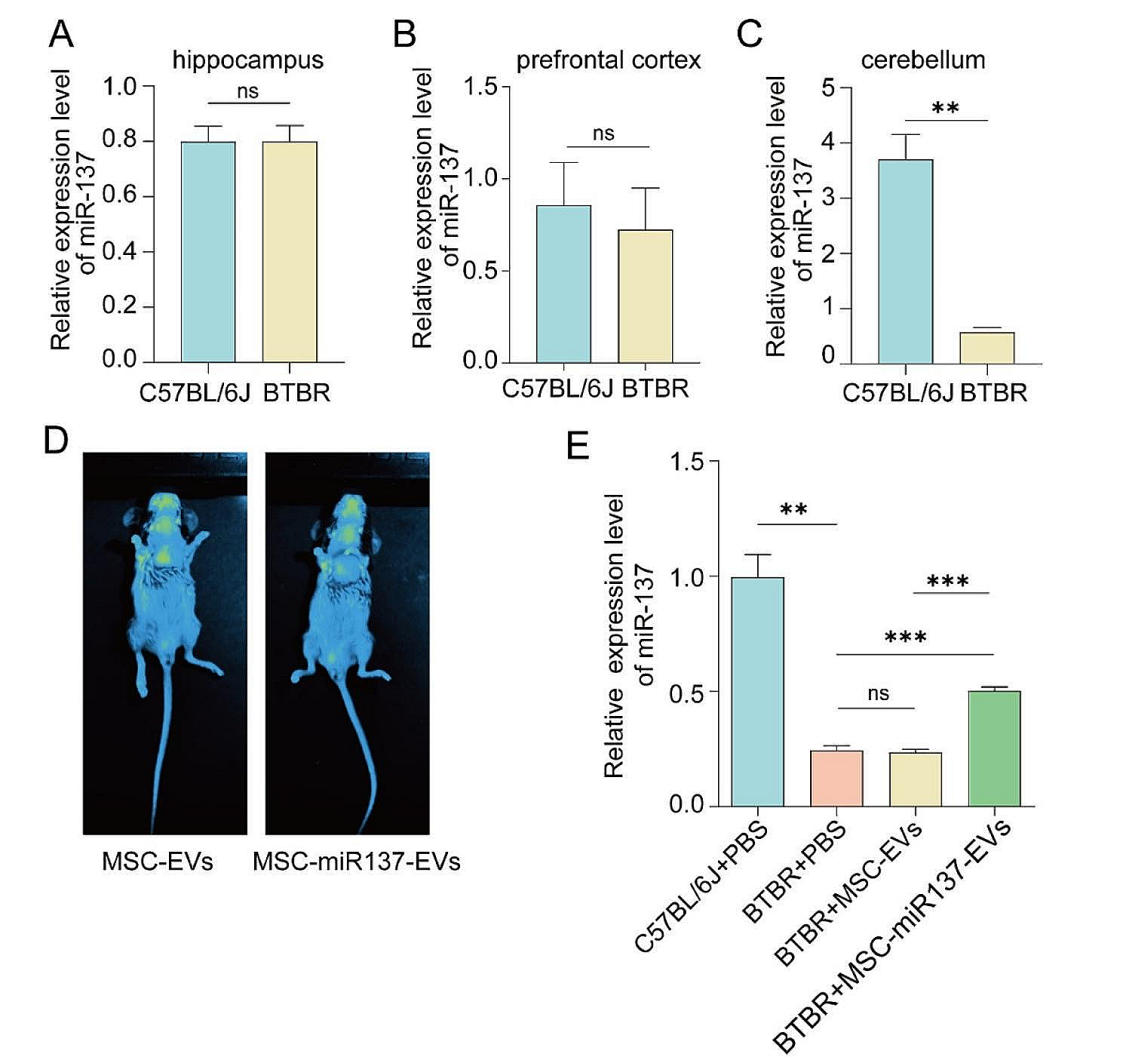
miR-137 was efficiently delivered by MSC-EVs into the brain. **(A–C)** Relative qRT-PCR analysis was performed to assess the reduced expression of miR-137 in the brain tissue of BTBR mice. ns *P* ≥ 0.05, **P < 0.01 versus the C57BL/6J group using Student’s *t* test. **(D)** In vivo imaging illustrating the distribution of DiR-labeled MSC-EVs and MSC-miR137-EVs in mice. **(E)** Relative qRT-PCR analysis of miR-137 levels in the mouse cerebellum after injection of MSC-EVs or MSC-miR137-EVs. ns *P* ≥ 0.05, **P < 0.01, ***P < 0.001 using one-way ANOVA followed by the Holm–Sidak post hoc multiple comparison test

To investigate the regions of decreased miR-137 expression in the BTBR mouse brain, relative qRT-PCR analysis was performed. As shown in Fig. 2A–C, the expression of miR-137 in the cerebellar tissue of BTBR mice was lower compared to that in control mice, while no significant difference was observed in the hippocampus and prefrontal cortex. To validate the delivery efficacy of EVs to the brain, 1,1′-dioctadecyl-3,3,3′,3′-tetramethylindotricarbocyanine iodide (DiR)-labeled EVs were monitored using an in vivo imaging system (IVIS) 6 h post-intranasal administration at a dosage of 200 µg (Fig. 2D). The in vivo delivery efficacy of miR-137 by MSC-EVs was then evaluated using relative qRT-PCR analysis (Fig. 2E).

### MSC-EVs-mediated delivery of miR-137 alleviated autism-like behaviors in BTBR mice

**Fig. 3:**
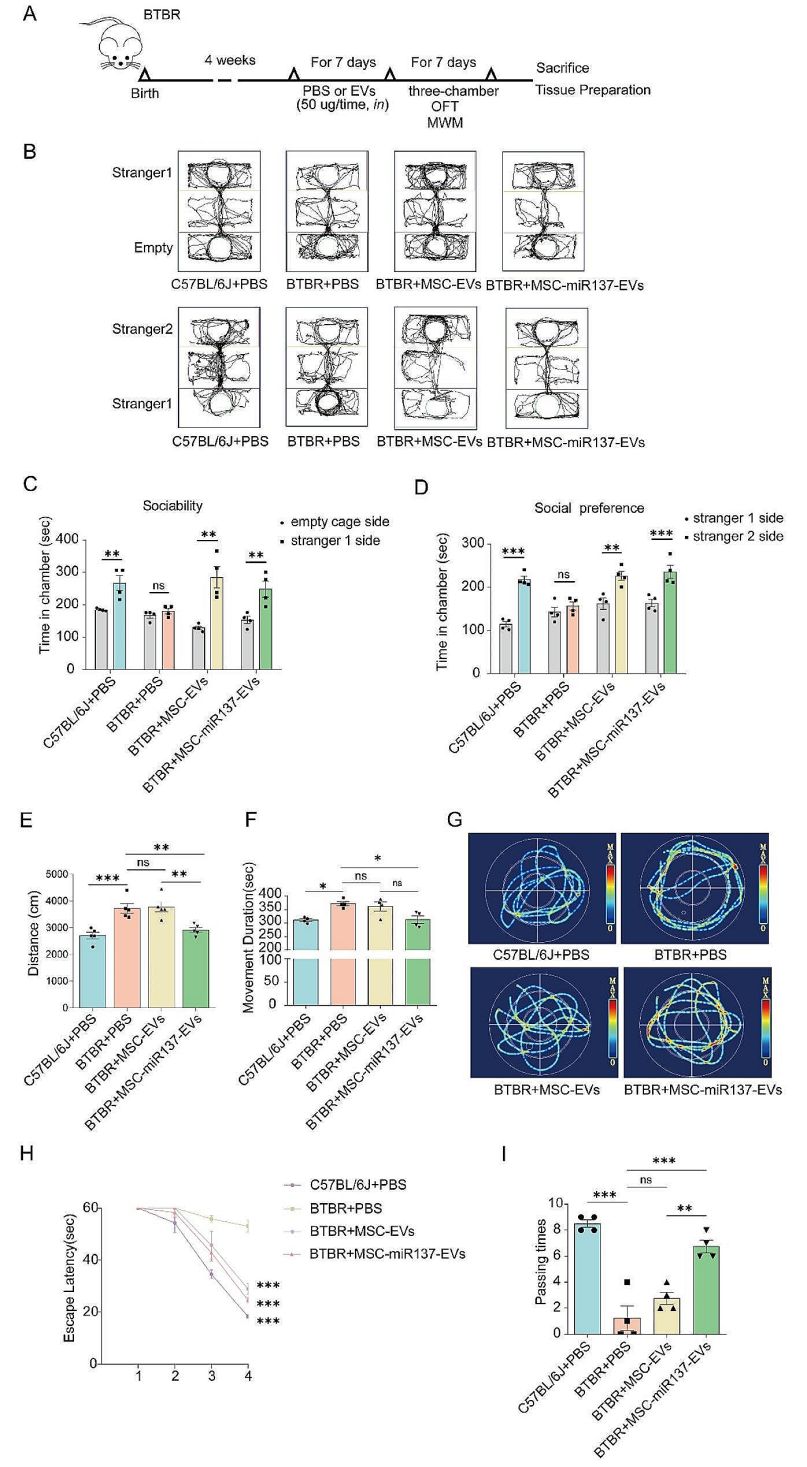
Effects of miR-137 and MSC-EVs on autism-like behaviors in BTBR mice. **(A)** Schematic representation illustrating the experimental procedure involving EVs and the subsequent behavioral studies conducted. **(B–I)** MSC-EVs and MSC-miR137-EVs relieved autism-like behaviors in BTBR mice, as measured by the three-chamber test **(B–D)**, OFT **(E–F)** and MWM **(G–I)**. ns *P* ≥ 0.05, *P < 0.05, **P < 0.01, ***P < 0.001 using one/two-way ANOVA followed by the Holm–Sidak post hoc multiple comparison test

The BTBR mice were intranasally administered MSC-EVs or MSC-miR137-EVs for 7 days. Next, the three-chamber test, open field test (OFT), and Morris water maze (MWM) test were conducted to evaluate the social ability, anxiety level, and learning and memory abilities of the spatial position and directional senses, respectively (Fig. 3A). As shown in Fig. 3B–I, BTBR mice administered MSC-EVs and MSC-miR137-EVs exhibited evidence of ameliorated autism-like behaviors.

In this study, the three-chamber test was employed to evaluate the social behaviors of mice. During the initial stage of the social ability test **(**Fig. 3B, C**)**, B6 mice showed a preference for interacting with Stranger 1 (S1), whereas BTBR mice showed no preference between interacting with S1 or the empty cage (E), indicating an impairment in the social abilities of the BTBR mice. However, BTBR mice in the MSC-EVs and MSC-miR137-EVs intervention groups exhibited enhanced social abilities post-intervention. In the social preference test stage **(**Fig. 3B, D**)**, B6 mice favored interaction with Stranger 2 (S2). Conversely, BTBR mice showed no preferential interaction with either S1 or S2, highlighting a compromised social preference. Yet, post-intervention with MSC-EVs and MSC-miR137-EVs, an improvement in the social preference of BTBR mice was observed.

In the OFT, compared to B6 mice, BTBR mice exhibited longer movement distances and durations of activity in the open field. However, BTBR mice in the MSC-miR137-EVs intervention group exhibited reduced movement distances and durations of activity relative to the BTBR mice. These results suggested an improvement in anxiety levels within the MSC-miR137-EVs group (Fig. 3E, F).

A five-day MWM experiment was conducted for each group of mice. During the learning phase, the BTBR mice demonstrated lower latency in finding the platform compared to B6 mice. In the test stage, the BTBR mice crossed the platform fewer times than B6 mice, indicating impairments in learning and memory. In comparison to the BTBR group, the mice in the MSC-EVs and MSC-miR137-EVs intervention groups exhibited quicker decreases in latency during the learning phase. Additionally, the test phase revealed an increase in the number of intervention group mice entering the platform area compared to the BTBR group **(**Fig. 3G–I**)**. These findings suggested that MSC-miR137-EVs has the potential to improve cognitive impairments related to spatial and directional learning and memory in BTBR mice.

### MSC-EVs-mediated delivery of miR-137 regulated TLR4/NF-κB to attenuate neuroinflammation

**Fig. 4:**
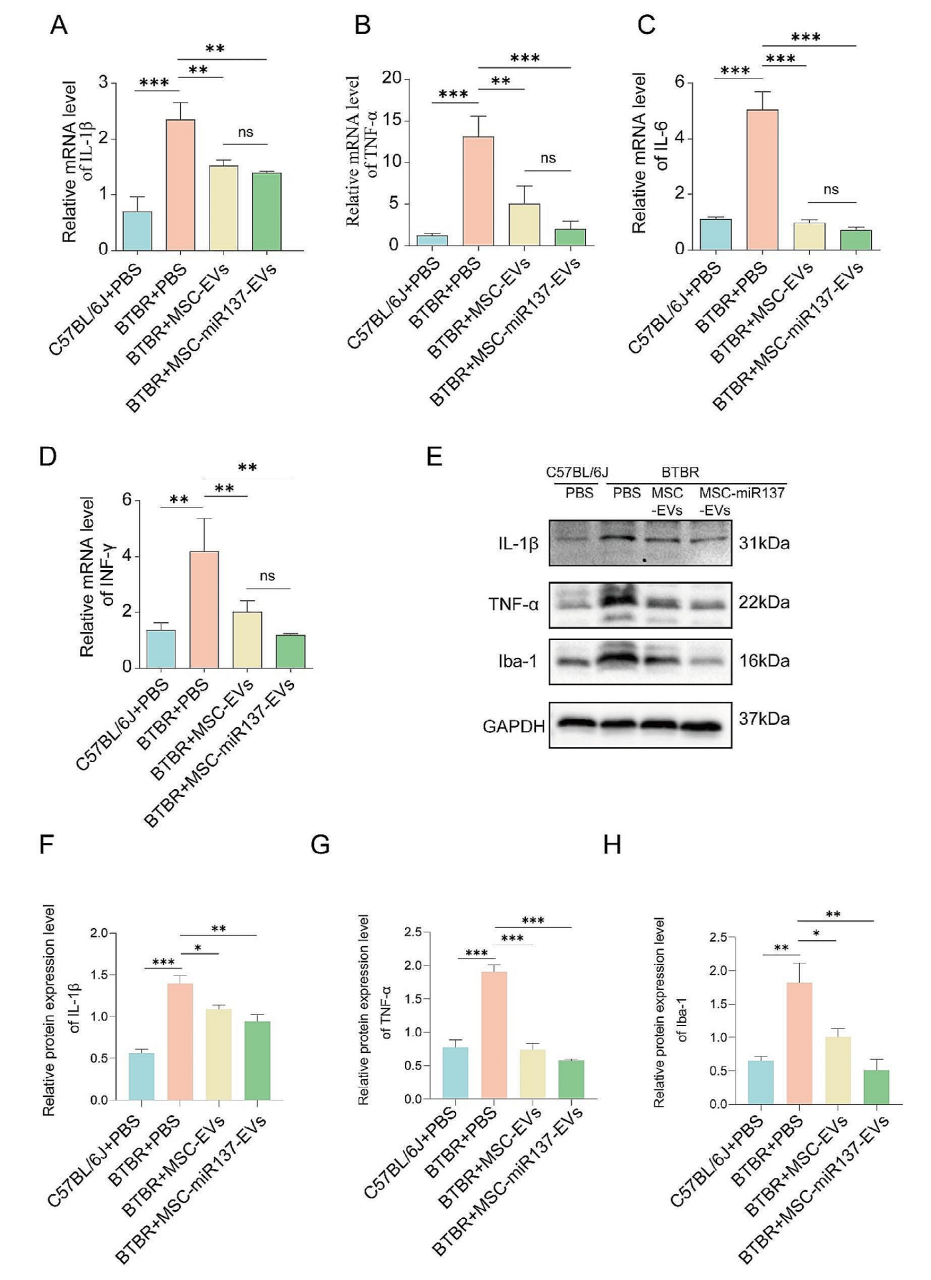
MSC-EVs-mediated delivery of miR-137 to attenuate neuroinflammation. **(A–D)** Expression levels of IL-1β **(A)**, TNF-α **(B)**, IL-6 **(C)**, and INF-γ **(D)** were assessed by relative qRT-PCR analysis in the cerebellum following administration of MSC-EVs and MSC-miR137-EVs in BTBR mice. ns *P* ≥ 0.05, **P < 0.01, ***P < 0.001 using one-way ANOVA followed by the Holm–Sidak post hoc multiple comparison test. **(E–H)** Western blot analysis was conducted to assess the levels of IL-1β, TNF-α, and Iba-1 following the administration of MSC-EVs and MSC-miR137-EVs in BTBR mice. ns *P* ≥ 0.05, *P < 0.05, **P < 0.01 using one-way ANOVA followed by the Holm–Sidak post hoc multiple comparison test

Immunofluorescence detection revealed that the expression of microglia marker ionized calcium-binding adaptor molecule 1 (Iba-1) in the cerebellums of BTBR mice was elevated compared to that in the control mice, indicative of neuroinflammation in the cerebellums of BTBR mice (Figure S1). To assess the ability of MSC-EVs and miR-137 to attenuate neuroinflammation, we evaluated the levels of pro-inflammatory factors in the cerebellum after 7-day EVs administration. First, the mRNA expression levels of interleukin-1β (IL-1β), IL-6, TNF-α, and interferon (INF)-γ and the protein expression levels of Iba-1, IL-1β, and TNF-α were increased in BTBR cerebellums compared to that in control mice cerebellums, indicating significant neuroinflammation and microglia activation in the BTBR cerebellum (Fig. 4A–D). After a 7-day injection, notable decreases were observed in the expression levels of Iba-1, IL-1β, and TNF-α in the BTBR cerebellums of both the MSC-EVs and the MSC-miR137-EVs groups compared to the control group. Remarkably, the reduction was more pronounced in the MSC-miR137-EVs group than in the MSC-EVs group. These observations suggested that MSC-EVs and miR-137 could ameliorate neuroinflammation in BTBR mice (Fig. 4E–H).

**Fig. 5:**
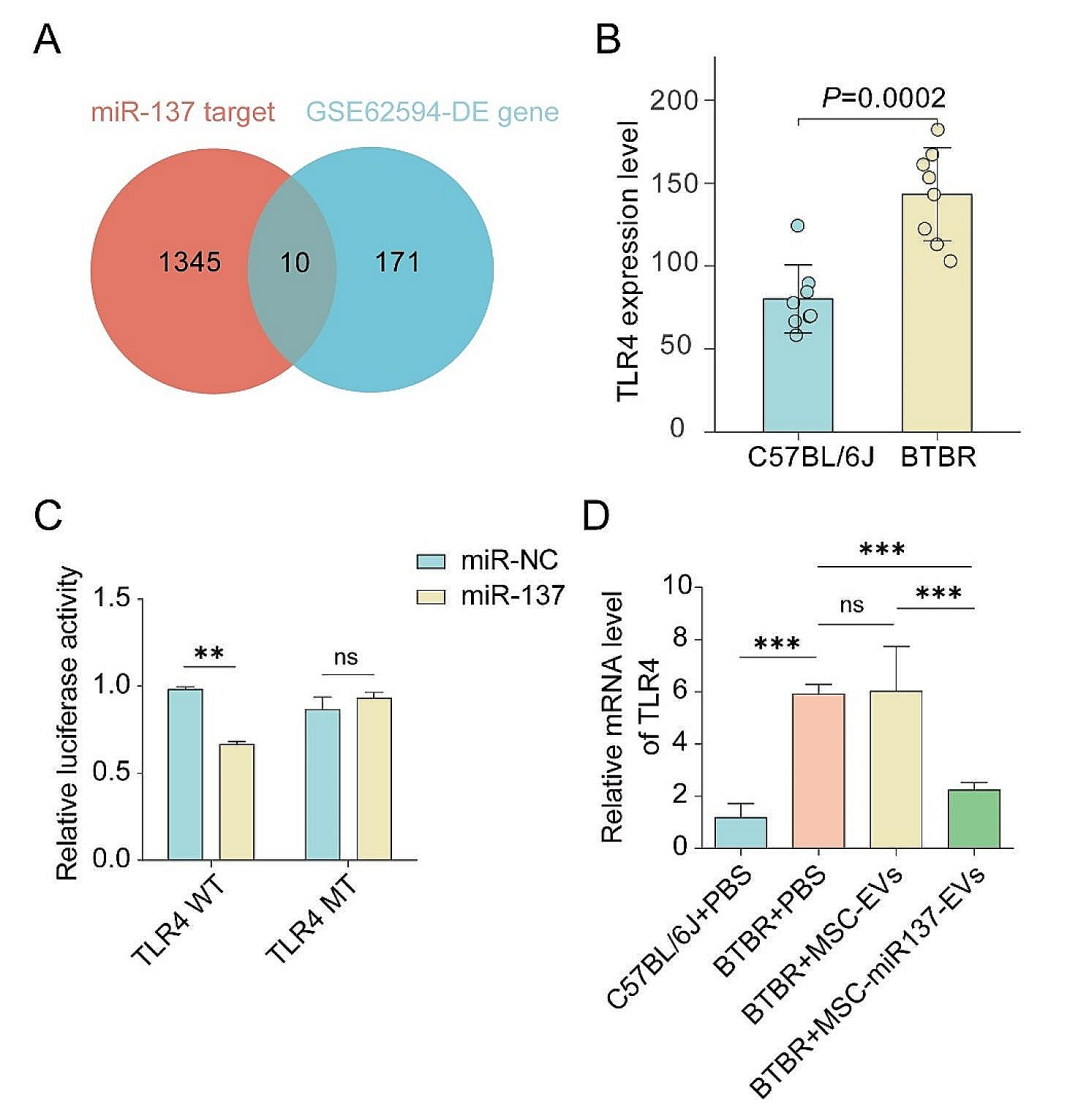
miR-137 bound to and downregulated the expression of TLR4. **(A–B)** The miRNA–mRNA interaction was predicted using the Starbase public databases, and the GSE62594 dataset was analyzed to examine differences in TLR4 expression in the cerebellum between C57BL/6J and BTBR mice. **(C)** Dual-luciferase assay was performed to determine that miR-137 targets TLR4. ns *P* ≥ 0.05, **P < 0.01 versus the control group using one-way ANOVA followed by the Holm–Sidak post hoc multiple comparison test. **(D)** qRT-PCR analysis of TLR4 expression in the cerebellums of BTBR mice after EVs were introduced. ns *P* ≥ 0.05, ***P < 0.001 using one-way ANOVA followed by the Holm–Sidak post hoc multiple comparison test

To elucidate the mechanism through which miR-137 attenuates inflammation in BTBR mice, we initially predicted the target genes of miR-137 utilizing the publicly available Starbase database (https://starbase.sysu.edu.cn/starbase2). Then, we analyzed the gene expression profiles within the GSE62594 dataset of the GEO database, which references cerebellum expression in B6 and BTBR mice, and screened the differential genes that were upregulated in the cerebellums of BTBR mice. Further, the genes related to ASD and neuroinflammation within the list of upregulated differentially expressed genes were screened through literature review. Ultimately, the specific candidate gene, TLR4, of miR-137 in the BTBR cerebellar tissue was identified by integrating the list of miR-137 target genes with genes ascertained from the GEO database. Subsequent experimental verification was conducted to verify these findings (Fig. 5A, B, Table S1).

To evaluate the binding of miR-137-3p with TLR4, the wild-type 3′-untranslated region (UTR) sequence and the mutated sequence were cloned into the pmirGLO vector. The reporter constructs were co-transfected into HEK293T cells with miR-137-3p mimic or a mimic negative control (NC). A dual-luciferase assay revealed that miR-137-3p decreased pmirGLO-TLR4-WT activity by binding to the target sequence. Furthermore, the inhibition of luciferase was abolished after mutation of the binding site (Fig. 5C).

The TLR4 levels in the cerebellum were assessed using qRT-PCR analysis post-intranasal administration of EVs. As shown in Fig. 5D, compared with the control group, the level of TLR4 mRNA in the cerebellums of mice injected with MSC-miR137-EVs was reduced compared with that in the control group, though the expression of TLR4 in the cerebellums of mice in the MSC-EVs group did not change significantly. These findings suggested that MSC-EVs facilitated the delivery of miR-137 into the mouse brain via intranasal administration.

**Fig. 6:**
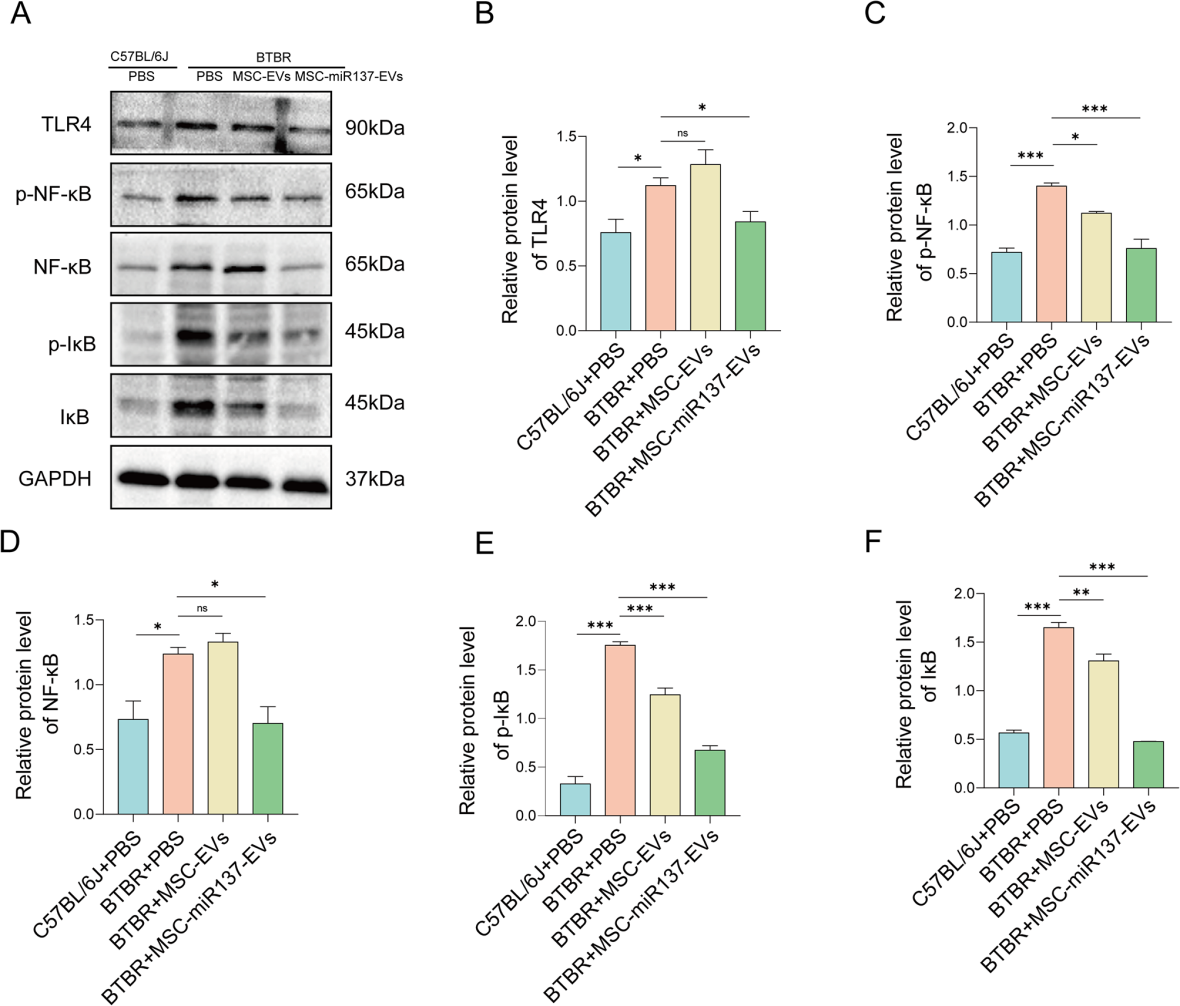
MSC-EVs-mediated delivery of miR-137 regulated the TLR4/NF-κB pathway. **(A)** Western blot analysis of the expression of members of the TLR4/NF-κB signaling pathway in the cerebellums of BTBR mice after the introduction of EVs. **(B–F)** Statistical results of the expression of members of the TLR4/NF-κB signaling pathway in the cerebellums of BTBR mice after the introduction of EVs. ns *P* ≥ 0.05, *P < 0.05, **P < 0.01, ***P < 0.001 using one-way ANOVA followed by the Holm–Sidak post hoc multiple comparison test. All datas are presented as the mean ± SEM of three independent experiments

The TLR4 expression level was higher in BTBR mice than in B6 mice, and the protein expression levels of phosphorylated NF-κB (p-NF-κB), NF-κB, phosphorylated inhibitor of NF-κB (p-IκB), and IκB were also higher in BTBR mice than in B6 mice, indicating that the TLR4/NF-κB pathway was over-activated in the cerebellums of BTBR mice. One week after the administration of EVs, compared with the control group, the TLR4 protein expression in the cerebellums of BTBR mice in the MSC-miR137-EVs group was reduced, indicating that the activation of the TLR4/NF-κB pathway had also decreased. In the MSC-EVs group, the TLR4 expression level had not decreased, but the activation of the NF-κB pathway was reduced (Fig. 6A–F).

### MSC-miR137-EVs inhibited mouse microglial activation and neurotoxicity in vitro

**Fig. 7:**
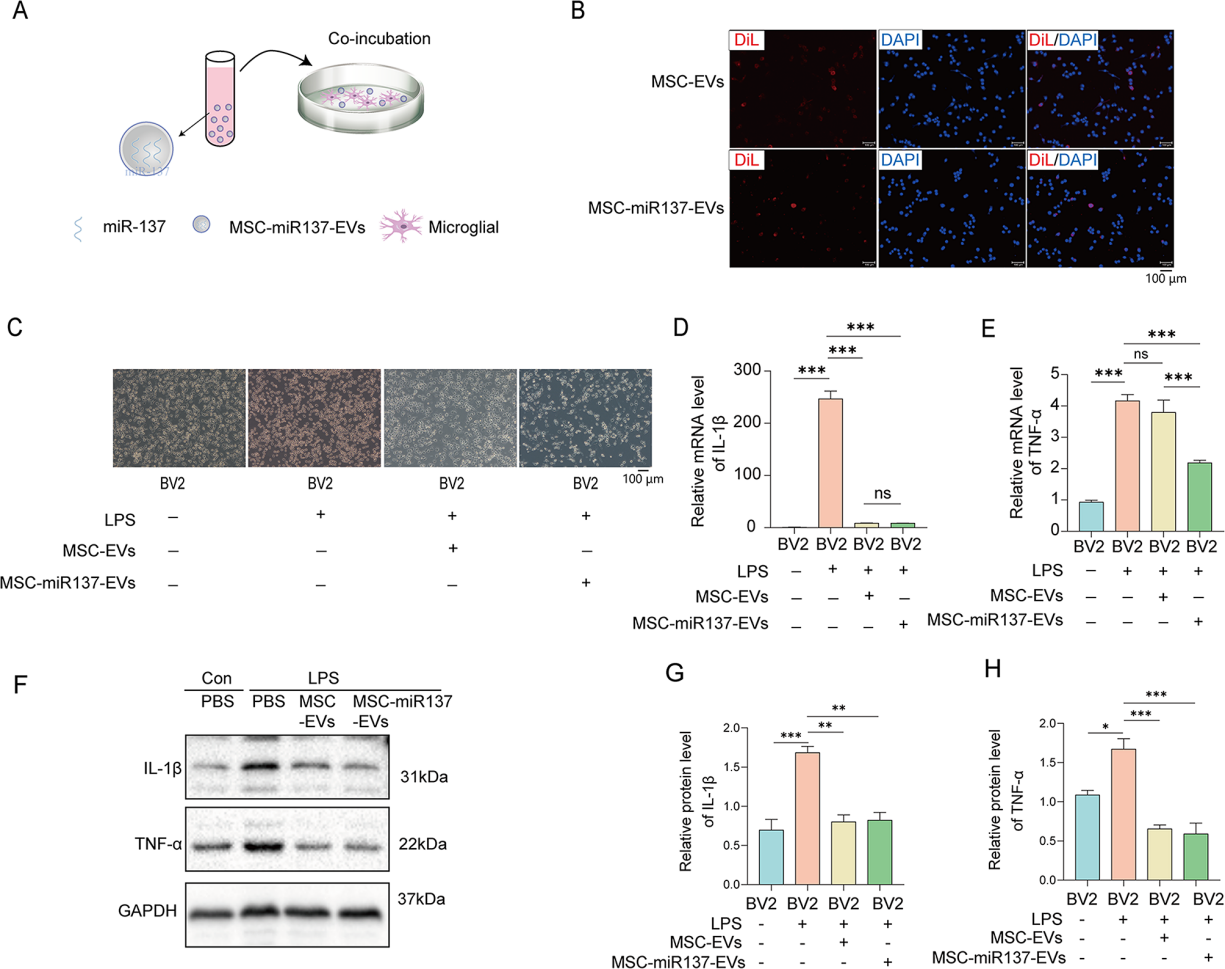
MSC-miR137-EVs inhibited mouse microglial activation in vitro. **(A)** Schematic illustration of the incorporation and uptake of engineered EVs into mouse microglial BV2 cells. **(B)** Fluorescence images depicting BV2 cells following their incubation with DiI-labeled EVs. Scale bar: 100 μm. **(C)** Morphological alterations in BV2 cells were visually examined under an optical microscope following incubation with either MSC-EVs or MSC-miR137-EVs. **(D–H)** The expression levels of IL-1β **(D)** and TNF-α **(E)** were quantified by relative qRT-PCR and Western blot analysis **(F–H)** in mouse microglia co-incubated with MSC-EVs or MSC-miR137-EVs with or without LPS treatment. ns *P* ≥ 0.05, **P < 0.01, ***P < 0.001 using one-way ANOVA followed by the Holm–Sidak post hoc multiple comparison test

To verify the inhibition of inflammation by MSC-EVs and miR-137 in vitro, MSC-EVs or MSC-miR137-EVs were co-incubated with lipopolysaccharide (LPS)-activated BV2 microglial (LPS-BV2) cells (Fig. 7A). MSC-EVs and MSC-miR137-EVs labeled with 2-[3-(1,3-dihydro-3,3-dimethyl-1-octadecyl-2 H-indol-2-ylidene)-1-propen-1-yl]-3,3-dimethyl-1-octadecyl-3 H-indolium, monoperchlorate (DiI) were internalized by BV2 cells at 3 h (Fig. 7B). To investigate the effect of EVs on the LPS-induced activation of BV2 cells, after treatment with EVs (50 µg) for 48 h, we first evaluated their morphological changes. As shown in Fig. 7C, resting microglia were spindle-shaped, with small cell bodies with long processes. After LPS treatment, microglia showed a pro-inflammatory M1 morphology, characterized by big cell bodies with short processes. However, the LPS-induced morphological changes in BV2 cells were attenuated after treatment with MSC-EVs and MSC-miR137-EVs. Then, we performed qRT-PCR and Western blot to detect the production of pro-inflammatory factors in BV2 cells. The results revealed that MSC-EVs and miR-137 could reduce the LPS-induced increases in IL-1β and TNF-α in microglia (Fig. 7D–H). These results suggested that both MSC-EVs and miR-137 can reverse LPS-induced microglia morphological changes and inflammatory factor production.

**Fig. 8:**
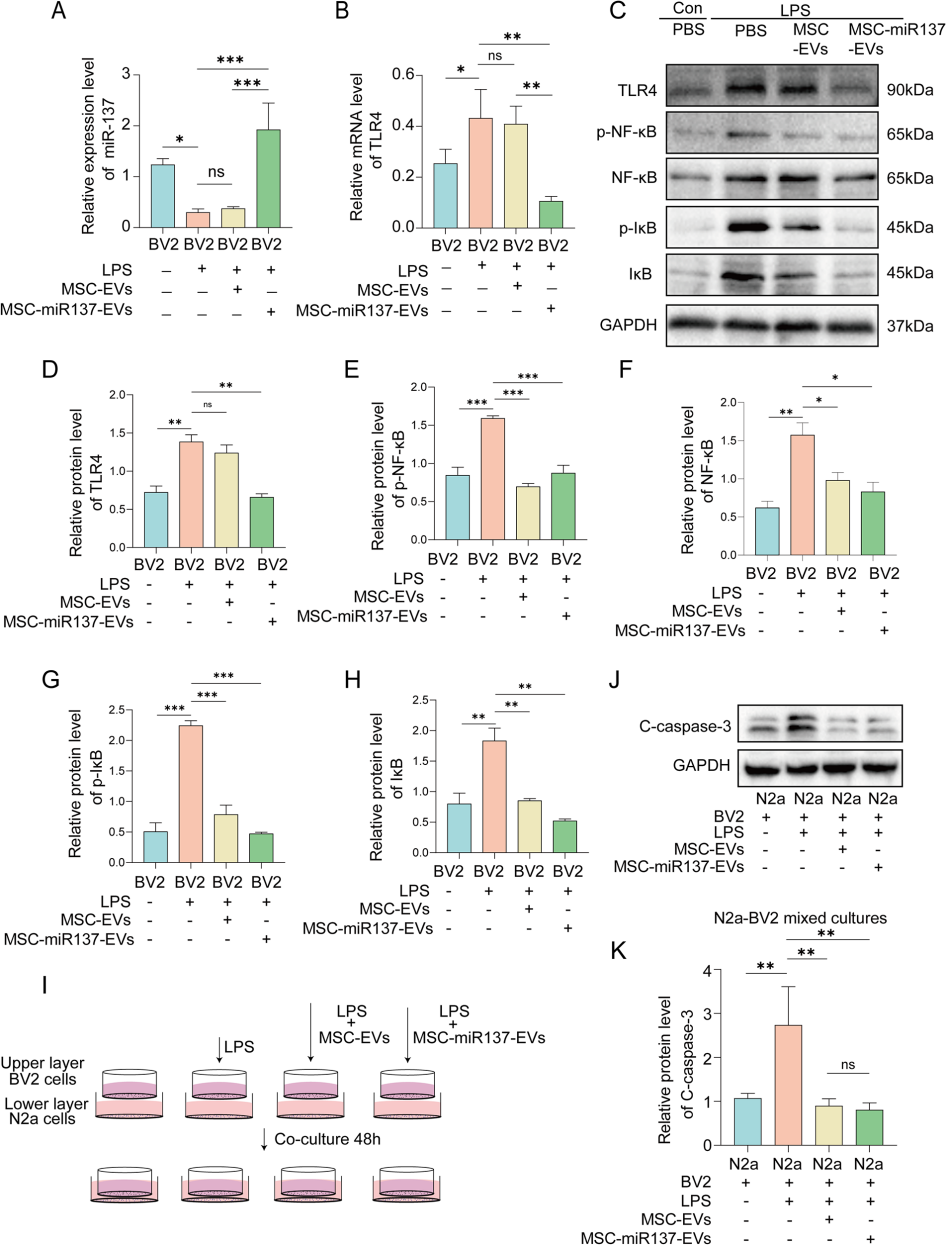
MSC-miR137-EVs inhibited mouse microglial activation and neurotoxicity by the TLR4/NF-κB pathway in vitro. (A–B) Relative qRT-PCR analysis was conducted to assess the levels of miR-137 and TLR4 in BV2 cells following co-incubation with MSC-EVs or MSC-miR137-EVs. ns *P* ≥ 0.05, *P < 0.05, ***P < 0.001 using one-way ANOVA followed by the Holm–Sidak post hoc multiple comparisons test. **(C–H)** Western blot analysis of the expression of members of the TLR4/NF-κB signaling pathway in LPS-induced BV2 cells after co-culture with EVs. ns *P* ≥ 0.05, *P < 0.05, **P < 0.01, ***P < 0.001 using one-way ANOVA followed by the Holm–Sidak post hoc multiple comparison test. **(I)** Schematic illustration of N2a cells co-incubated with BV2 cells co-cultured with MSC-EVs or MSC-miR137-EVs with or without LPS treatment. **(J–K)** Western blot analysis of cleaved caspase-3 levels in N2a cells after co-incubation with LPS-BV2 cells co-cultured with MSC-EVs or MSC-miR137-EVs with or without LPS treatment. ns *P* ≥ 0.05, **P < 0.01 using one-way ANOVA followed by the Holm–Sidak post hoc multiple comparison test

Compared with the control group, the miR-137 level in LPS-induced BV2 cells decreased, and the administration of MSC-miR137-EVs increased the level of miR-137 in microglia after co-incubation with LPS-BV2 cells (Fig. 8A). Meanwhile, we evaluated the TLR4 mRNA expression levels in LPS-induced BV2 cells after co-culture with EVs by qRT-PCR analysis, which revealed that TLR4 mRNA levels were decreased in BV2 cells co-cultured with MSC-miR137-EVs compared with the control group (Fig. 8B).

After 48 h of co-culture of BV2 cells with EVs, we measured the TLR4 expression and the activation of the TLR4/NF-κB pathway in BV2 cells (Fig. 8C–H). In LPS-induced BV2 cells, the TLR4 expression level was higher than that in control cells, and the protein levels of p-NF-κB, NF-κB, p-IκB, and IκB were also higher than in uninduced BV2 cells, indicating that the TLR4/NF-κB pathway was over-activated in the LPS-induced BV2 cells. However, in the MSC-miR137-EVs group, the activation level of the TLR4/NF-κB pathway was decreased. Surprisingly, in the MSC-EVs group, the TLR4 expression level was not different, but the activation of the NF-κB pathway was reduced. In conclusion, miR-137 could alleviate the activation of the TLR4/NF-κB pathway in LPS-induced BV2 cells, and MSC-EVs could also decrease the activation level of the NF-κB pathway.

To further elucidate whether microglial polarization could affect neuronal apoptosis, BV2 microglia and N2a cells were co-cultured using a Transwell system, as shown in Fig. 8I. Briefly, BV2 cells in the upper chamber were divided into the following groups: control, LPS, LPS with MSC-EVs, and LPS with MSC-miR137-EVs. N2a cells were seeded in the lower chamber. The Western blot analysis revealed that the level of the apoptosis-associated protein cleaved caspase-3 was increased in the N2a cells in the LPS group, but the levels were observably decreased in the MSC-EVs and MSC-miR137-EVs groups (Fig. 8J, K). In brief, these results indicated that MSC-miR137-EVs could efficiently convert microglia from the proinflammatory M1 phenotype and restrain neuronal apoptosis, which may be related to the microglia phenotype conversion.

### MSC-EVs alleviate neuroinflammation via the miR-146a-5p/TRAF6 pathway

**Fig. 9:**
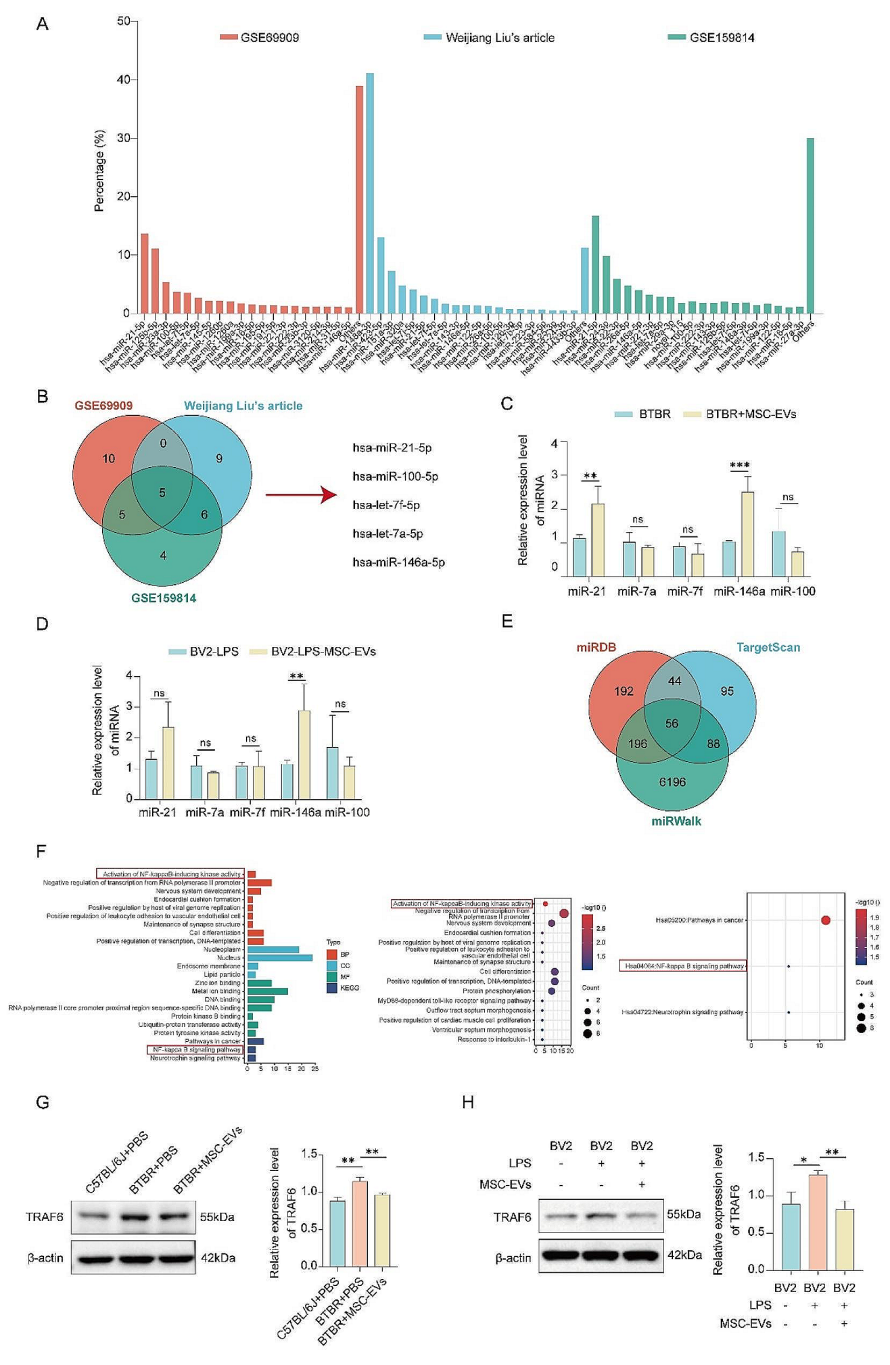
MSC-EVs alleviated neuroinflammation via the miR-146a-5p/TRAF6 pathway. **(A)** Analysis of miRNA abundance in MSC-EVs conducted utilizing datasets GSE69909 and GSE159814 from the GEO database, complemented by sequencing data from a publication by Weijiang Liu. **(B)** Venn diagram of the intersection of the 20 most abundant miRNAs identified from three different datasets and the overlapping miRNAs. **(C)** Relative miRNA expression in the cerebellum tissue of BTBR mice pretreated with MSC-EVs, relative to the PBS group. **(D)** Relative miRNA expression in LPS-BV2 cells pretreated with MSC-EVs, relative to the PBS group. **(E)** Venn diagram of the intersection of the mRNA targets identified from three miRNA databases and the overlapping genes. **(F)** GO and KEGG analysis of the predicted mRNA targets for miR-146a-5p in the three datasets. **(G)** Western blot analysis of the relative expression levels of TRAF6 in the cerebellum tissue of mice. **P < 0.01. Data are the mean ± SEM and were analyzed using one-way ANOVA followed by the Holm–Sidak post hoc multiple comparison test. **(H)** Western blot analysis of the relative expression levels of TRAF6 in BV2 cells. *P < 0.05, **P < 0.01. Data are the mean ± SEM and were analyzed using one-way ANOVA followed by the Holm–Sidak post hoc multiple comparison test

Previous research has demonstrated that the predominant functional RNA components in exosomes are miRNAs, which can be effectively transmitted to other cells to perform various function through exosomal integration [19]. miRNAs, one of the most important cargos in exosomes, are transferred to other cells and play important roles in regulating inflammatory diseases, such as osteoarthritis [20] and inflammatory pain [21]. Therefore, to investigate the possible mechanism by which MSC-EVs influence neuroinflammation of ASD, we analyzed the top 20 highly enriched miRNAs in the GSE69909 and GSE159814 public datasets and an additional file from a report by Weijiang Liu (Fig. 9A). The five overlapping miRNAs in MSC-EVs from the above three datasets, hsa-miR-21-5p, hsa-miR-100-5p, hsa-let-7f-5p, hsa-let-7a-5p, and hsa-miR-146a-5p, were screened (Fig. 9B). Compared with that in the PBS group, the relative expression levels of miR-146a-5p in the cerebellum tissue of BTBR mice and LPS-BV2 cells pretreated with MSC-EVs were both significantly increased, as shown by qRT-PCR (Fig. 9C, D). Then, the miRWalk, miRBD, and TargetScan databases were used to predict the target genes of miR-146a-5p. There were 56 overlapping genes in the three public databases (Fig. 9E). Gene Ontology (GO) terms and Kyoto Encyclopedia of Genes and Genomes (KEGG) pathway enrichment bioinformatics analysis revealed that the target genes of miR-146a-5p were highly involved in the activation of NF-κB-inducing kinase activity, negative regulation of transcription from the RNA polymerase II promoter, nervous system development, and positive regulation of viral genome replication by the host (Fig. 9F). Intriguingly, the targeted genes of miR-146a-5p were involved in the NF-κB signaling pathway, which is well-known to activate neuroinflammation [22]. Meanwhile, TRAF6 was one of the most relevant target genes of miR-146a-5p in the NF-κB signaling pathway. Western blot analysis revealed that TRAF6 protein expression was significantly decreased in the cerebellum tissue of BTBR mice and LPS-BV2 cells treated with MSC-EVs compared with those in BTBR mice treated with PBS and LPS-BV2 cells (Fig. 9G, H). Generally, these data suggested that MSC-EVs alleviated neuroinflammation in LPS-treated BV2 cells and BTBR mice via the miR-146a-5p/TRAF6 pathway.

## Methods

### Cells culture

The primary human umbilical cord tissue derived mesenchymal stem cells (hUC-MSC) isolated from human umbilical cord were purchased from the Nanjing Drum Tower Hospital. The human embryonic kidney 293T cells (HEK-293T cells) were obtained from Department of Histology and Embryology, Harbin Medical University. BV2 murine microglial cells were obtained from Department of Children’s and Adolescent Health, Harbin Medical University. The mouse neuroblastoma cell line, Neuro 2a (N2a) cells were obtained from Department of Neurobiology, Harbin Medical University. In addition, FBS for EVs production was depleted of bovine EVs by ultracentrifugation at 160,000×g for 16 h at 4 °C using an CP100NX ultracentrifuge (Hitachi, Japan). All cells were cultured in Dulbecco’s modified Eagle’s medium (DMEM, GIBCO, USA) supplemented with 10% fetal bovine serum (FBS, GIBCO, USA), 1% penicillin-streptomycin (Beyotime, China), and kept in a humidified incubator (5% CO_2_, 37 °C).

### Transfection and preparation of EVs

The miR-137 overexpressed lentivirus was constructed by GeneChem Company, China. Briefly, the miR-137 coding sequence was cloned into the hU6-MCS-Ubiquitin-EGFP-IRES-puromycin lentivirus vector by using *Age*I and *Eco*RI restriction endonuclease (Figure S2). When MSC cells reached the third-sixth generation, the cells were seeded in six-well plate. Then, when the cells reached approximately 30% confluence, MSC cells were transfected with the miR-137 overexpressed lentivirus according to the manufacturer’s instructions. Next, MSC-EVs or MSC-miR137-EVs were prepared from the supernatant fluids of MSC cells or MSC-miR137 cells respectively by differential centrifugation. When MSC cells or MSC-miR137 cells reached the sixth-thirteenth generation, and were grown to 80–90% confluence, they were rinsed three times with phosphate-buffered saline (PBS) and switched to serum-free DMEM. The supernatants were harvested after 48 h to isolate EVs. Firstly, the supernatants were centrifuged at 300×g for 10 min and 2000×g for 10 min to eliminate cells and dead cells. Then, the supernatants were centrifuged at 10,000×g for 30 min to remove cellular debris. Next, the supernatant was centrifuged at 100,000×g for 60 min at 4 °C using an CP100NX ultracentrifuge (Hatichi, Japan). Subsequently, the pellet was resuspended in PBS and then ultracentrifuged again at 100,000×g for 60 min. All steps were performed at 4 °C. Finally, the resulting pellet was resuspended in PBS for further study.

### Characterization of EVs

Protein content of EVs was measured with BCA Protein Assay Kit (P0011, Beyotime, China) following the manufacturer’s protocol. The markers of purified EVs were verified by Western blotting (WB) analysis. During WB test, the following antibodies were used: CD9 (1:1000, #13174, Cell Signaling Technology, USA), CD63 (1:1000, ab134045, Abcam, UK), TSG101 (1:1000, ab125011, Abcam, UK), Calnexin (1:1000, #2679, Cell Signaling Technology, USA). The transmission electron microscopy (TEM) (H-7650, HITACHI, Japan) was used to observe the morphology of EVs. The nanoparticles were visualized and quantitated by Zetasizer Nano ZS90 Nanometer particle size meter (Malvern, UK) in suspension. The EVs were stored at -80 °C for following applications.

### qRT-PCR

The RNA was extracted from the cerebellum tissue of mice or BV2 cells using RNAiso Plus (Takara, Japan). Reverse transcription was then performed using the reaction tube mentioned above mixed with 2 µl of 2 × qRT Enzyme (Takara, Japan) and nuclease free water up to 10 µl of the final reaction volume. This reaction mixture was incubated at 37 °C for 15 min and then for 2 min at 85 °C. Next, the RT products (cDNA) obtained in the previous step were used as the template for qRT-PCR. The qRT-PCR reactions were carried out using SYBR Green qRT-PCR Master Mix (Q712-02, Vazyme, China) containing 1 µl of cDNA in a 10 µl final volume reaction with the following steps: 5 min at 95 °C followed by 45 cycles of 15 s at 95 °C, 30 s at 60 °C and 30 s at 72 °C, then followed 5 s at 95 °C, 1 min at 60 °C, 10 s at 95 °C and 30 s at 50 °C. The 2^−ΔΔ^Ct method was used to calculate the relative expression levels of mRNA. Primers were synthesized by Sangon Biotech (Shanghai, China). The primers used in this article are listed in table S2.

### EVs staining

The EVs samples were stained and purified based on the steps described in previous studies as follows [23]. The resuspended EVs protein concentration was 100 µg/ml and then stained with Dil (C1036, Beyotime, China) or DiR (D131031, Aladdin, USA) dyes at 37 °C for 15 min. Excess dye was bound with 10% EVs removal rate of fetal bovine serum. EVs were diluted with PBS and ultracentrifuged at 100,000×g for 1 h at 4 °C. The pellet was gently resuspended in 200 µl PBS.

### EVs uptake by BV2

A dose of 50 µg of MSC-EVs or MSC-miR137-EVs was resuspended in serum-free medium and added to BV2 cells. After incubation, cells were washed three times with PBS to remove excess EVs and to prepare for subsequent experiments. BV2 cells (2 × 10^4^ cells) were seeded in 35 mm discs. 24 h later, Dil-labeled control MSC-EVs or MSC-miR137-EVs were added to the cultured cells. Then, 3 h later (37 °C, 5% CO_2_), cells were stained with DAPI (Beyotime, China) and photographed with a Fluro microscope (ZEISS, Germany).

### In vivo imaging system (IVIS)

DiR-labeled MSC-EVs or MSC-miR137-EVs was used to investigate biodistribution in mice at the dose of 200 µg in this study. Then after 6 h, the intensity and distribution of fluorescence were recorded in vivo using an IVIS Spectrum Imaging System (Vilber Lourmat, France).

### Immunofluorescence staining

The mice were perfused with 100 ml PBS and then 25 ml 4% PFA and the brain tissues were isolated and cut into 10 μm thickness and were treated by 0.3% Triton X-100 (Beyotime, China) for 15 min and blocked with 10% normal goat serum (ZLI-9056, ZSGB-BIO, China) in 0.3% Triton X-100 for 1 h at room temperature. The sections were incubated with anti-Iba-1 (1:250, 019–19741, Wako Pure Chemicals) antibodies over night at 4 °C. After washing three times using PBS, the sections were incubated with Alexa 488-conjugated goat anti-rabbit IgG (1:1000, SA00013-2, Proteintech group, USA) for 1 h. The samples were washed three times with PBS and stained with an anti-quench agent containing DAPI. Images were taken with a laser confocal microscope (Nikon, Japan).

### Animals

C57BL/6J (B6) mice were purchased from the Beijing Vital River Laboratory Animal Technology Co., Ltd. BTBR T + Itpr3tf/J (BTBR) mice were bred at the Jackson Laboratory as a model for ASD. Mice were randomly assigned to different groups and maintained in a 12-h light/dark cycle with a temperature of 21 ± 1 °C; humidity of 55 ± 5%, and food and water were freely available. All mice procedures were approved by the ethnic committee of Harbin Medical University (Approval number: HMUIRB20210002).

### Intranasal delivery of EVs in animals

PBS, MSC-EVs or MSC-miR137-EVs were injected intranasally into BTBR mice. In brief, each mouse was injected with 50 µg of EVs every other day, four times in total. To determine the tissue distribution of miR-137 overexpression in vivo, the cerebellar tissue was collected and monitored the delivery efficiency through qRT-PCR. BTBR mice and control (B6 mice) were grouped to evaluate the improvement of miR-137 on autism behaviors. To explore the effect of miR-137 mediated by MSC-EVs on autism-like behaviors in BTBR mice, the mice were divided into four groups: C57BL/6J + PBS group, BTBR + PBS group, BTBR + MSC-EVs group and BTBR + MSC-miR137-EVs group.

### Behavioral tests

All behavioral tests were performed in a quiet and low-intensity environment and scored by the same researcher. Mice were moved to the test room at least 3 h prior to the behavioral test.

### Three-chamber test

The three-chamber device is a box with three chambers, each with an area of 20 cm × 40 cm. The three-chamber test was performed to detect social ability and social preference of mice. In the first adaptation phase, the test mouse was placed in the middle-chamber for 10 min. In the second phase of the sociability test, this was considered evidence of social behaviors when the test mice were within 2 cm of the cage or caged mice. Stranger mouse 1 (S1) was placed in a mouse cage in the left chamber. The empty cage was placed in the right chamber, while the test mice were placed in the central chamber. In addition, the movement and activity time in each box was recorded by a video camera for 10 min. This phase tested the preference of the test mice for contact with unfamiliar mice compared to the empty cage. The social preference test then began with the cage containing stranger mouse 1 (S1) being switched to the right chamber, while the cage containing mouse 2 (S2) was placed in the left chamber. The movements and activity times of the test mice were recorded by a video camera for 10 min similarly. This phase tested the preference of the test mice for contact with new, unfamiliar mice compared to familiar mice.

### Open field test (OFT)

The OFT was performed in a quiet field which was a 45 cm × 45 cm × 40 cm open box made of black polycarbonate and used to test the locomotor and anxiety-like exploratory behaviors in mice. The test mouse was placed in the center area for simultaneous imaging and timing. After a 5 min adaptation phase, each mouse was allowed to explore the chamber for 10 min and the movement time, distance and trajectory of the mice were recorded using the SMART 3.0 experiment system.

It is a commonly used experiment to evaluate the anxiety of mice by detecting their spontaneous activity behaviors and exploratory behaviors. The movement distance and movement time of mice are regarded as the main data reflecting their anxiety behaviors. Within a certain period of time, the longer the movement distance and movement time of mice reflect the more serious their anxiety behaviors.

### Morris water maze test (MWM)

The MWM test was used to assess the cognitive function of mice with respect to position and orientation. The experimental equipment used for this test was the WMT-100 Morris Water Maze Automated Analysis System with a 160 cm diameter flume carrying a far-infrared camera. The sink was divided into four quadrants, and an 8 cm × 8 cm platform was placed on the third quadrant. The liquid was made opaque with non-toxic white paint. The water surface was 1–2 cm above the platform. The experiment consisted of day 1–4 of training, which was performed twice a day. A training trial was accomplished when the mice found a platform within 60 s and stayed on the platform for 30 s after the trial when the mice climbed on the platform. On the fifth day, the spatial exploration ability was tested. The platform was displaced, the mice were placed in the first quadrant, and the number of times they traversed the original platform position within 60 s was recorded. Mice with cognitive deficits should traverse the platform less often in the exploration experiment.

### Western blotting

Lysates were harvested from cerebellum tissue of mice, BV2 cells or N2a cells in buffer containing RIPA (Applygen, China), protease inhibitor (MedChemexpress, USA), and phosphatase inhibitor (Applygen, China). Protein concentrations were determined using the BCA kit (Applygen, China), after which a total of 30 µg of protein were separated by sodium dodecyl sulphate-polyacrylamide gel electrophoresis (SDS-PAGE) and then transferred onto polyvinylidene fluoride membranes (PVDF, Millipore, USA). The PVDF membranes were blocked with 5% non-fat milk at room temperature for 1 h, Next, the membranes were incubated with primary antibody solution at 4 °C overnight. The primary antibodies includes Anti-CD9 (1:1000, #13174, Cell Signaling Technology, USA), Anti-CD63 (1:1000, ab134045, Abcam, UK), Anti-TSG101 (1:1000, ab125011, Abcam, UK), Anti-Calnexin(1:1000, #2679, Cell Signaling Technology, USA), Anti-TLR4 (1:1000, 19811-1-AP, Proteintech group, USA), Anti-p-IKBα (1:500, sc-8404, Santa Cruz Biotechnology, USA), Anti-IKBα (1:500, sc-1643, Santa Cruz Biotechnology, USA), Anti-p-NF-κB (1:500, sc-135769, Santa Cruz Biotechnology, USA), Anti-NF-κB (1:500, sc-8008, Santa Cruz Biotechnology, USA), Anti-IL-1β (1:1000, D220820, Sangon Biotech, China), Anti-TNF-α (1:1000, sc-52746, Santa Cruz Biotechnology, USA), Anti-Iba1 (1:1000, 17168-1-AP, Proteintech group), Anti-Cleaved Caspase-3 (1:2000, 20260-1-AP, Proteintech group), Anti-TRAF6 (1:1000, BS3684, bioworlde, China), Anti-β-actin (1:10000, 81115-1-RR, Proteintech group, USA), or Anti-GADPH (1:10000, 60004-1-AP, Proteintech group) overnight at 4 °C. After washing three times with TBST, the membranes were incubated with secondary antibody (1:10000, Boster, China) for 2 h. Proteins on the membranes were detected using the ECL Plus kit (MA0186-1, meilunbio, China) and bands were detected and quantified using Image-Pro Plus software by chemiluminescence (BIO-RAD, USA). The protein band intensity was quantified by ImageJ software and exhibited as relative density to GAPDH.

### Dual-luciferase reporter assay

PmirGLO/TLR4-3′-UTR WT and pmirGLO/TLR4-3′-UTR MT reporter plasmids were constructed in advanced according to the binding sites, and the binding sites of miR-137 and TLR4 were predicted through the Starbase database. HEK-293T cells were seeded into six-well plates, and according to the manufacturer’s instruction, when the confluence of HEK-293T cells reached to 70-80%, the cells were transiently co-transfected with miR-137 mimic or control mimic together with PmirGLO/TLR4-3′-UTR WT and pmirGLO/TLR4-3′-UTR MT reporter plasmids using Golden-Tran-DR (DR149260005-S, Golden Transfer Science and Technology, China). After 48 h, Dual-luciferase Reporter Assay System (E1910, Promega, USA) was used to detect firefly and Renilla luciferase activities and recorded using GloMax 96 Microplate Luminometer (Promega, USA).

### BV2 and N2a coculture system

BV2 cells were exposed to LPS (M1 phenotype inducer, 1 µg/mL, Sigma-Aldrich, USA) for the imitation of BV2 cells M1 phenotype. To elucidate the influence of microglia polarization on the neurotoxicity, we co-cultured BV2 cells with N2a cells in a 0.4 μm pore size Transwell co-culture system (Corning, USA). BV2 cells were seeded in the upper chamber (1 × 10^5^/chamber), which were then treated with PBS, EVs (50 µg/mL) or LPS respectively. After LPS exposure, the inserts were gently rinsed three times and placed above the N2a cells (5 × 10^5^/well) in 24-well plates. N2a cells were collected after co-culture for 48 h for cell apoptosis analysis.

### Public data availability and bioinformatic analysis

The miRNA expression profile datasets of MSC-EVs were obtained from the Gene Expression Omnibus (GEO, https://www.ncbi.nlm.nih.gov/geo/) public database as GSE69909 [24] and GSE159814 [25], and additional file from Weijiang Liu’s article (https://stemcellres.biomedcentral.com/articles/10.1186/s13287-021-02159-2) [26]. Nextly, the top 20 miRNAs in above three datasets about MSC-EVs were selected. TargetScan [27], miRDB [28] and miRwalk [29] were used to predict the target genes of miRNAs enriched in MSC-EVs. The expression profiling datas of the cerebellum in B6 and BTBR mice were obtained from GSE62594 dataset of the GEO database [30]. The miR-137 expression datas of peripheral blood from ASD patients and controls were also obtained from the GEO public database as GSE89596 [31]. All predicted targets were subjected to GO and KEGG analysis by DAVID online [32].

### Statistical analysis

Statistical analysis was performed by GraphPad Prism software. All datas were presented as mean ± SEM. Significance was assessed with a two-tailed Student’s *t* test for comparisons of two groups. One/two-way ANOVA followed by the Holm-Sidak test were used for multi-group (three or more) comparisons. The results were considered to be statistically significant if *P* < 0.05.

## Discussion

In this study, we successfully performed that MSC-EVs and MSC-miR137-EVs could ameliorate autism-like behaviors in BTBR mice. Mechanism studies revealed that miR-137 could target to TLR4 and alleviate the microglial activation, neuroinflammation and neurotoxicity through downregulating the TLR4/NF-κB signaling pathway. It was also relieved the inflammatory response of BV2 cells induced by LPS, and further reduced the apoptosis of N2a cells. Additionally, we demonstrated that MSC-EVs could also alleviate neuroinflammation and autism-like behaviors in BTBR mice via the miR-146a-5p/TRAF6 pathway.

In the initial segment of this study, we demonstrated that MSC-EVs could effectively deliver miR-137 to the mouse cerebellum. The therapeutic administration of MSC-miR137-EVs ameliorated the autism-like behaviors exhibited by BTBR mice. Moreover, miR-137 delivered by MSC-EVs reduced central nervous system (CNS) inflammation through the inhibition of microglial BV2 cell activation, in addition to the remission of microglial activation, neuroinflammation, and neurotoxicity. Consistent with previous reports, an augmented activation and quantity of microglial cells have been observed in the prefrontal cortices of individuals with ASD [33]. This abnormal neuroinflammatory activation potentially contributes to emotional disorders, behavioral abnormalities, and cognitive impairments [34, 35].

Current research on ASD has traditionally focused on the prefrontal cortex and hippocampus. However, our study highlights the importance of the cerebellum as a candidate brain region for ASD. Perets et al. revealed that MSC-EVs could be accumulated significantly in the cerebellum of BTBR mice as well, by utilizing gold nanoparticles [36, 37]. Additionally, we also found that MSC-EVs and MSC-miR137-EVs could reduce neuroinflammation in the cerebellum and alleviate autism-like behaviors in BTBR mice, underscoring the crucial role of the cerebellum in the pathological processes of ASD.

Our findings reveal that the reintroduction of miR-137 mitigates autism-like behaviors by targeting TLR4, subsequently regulating microglial activation via the NF-κB signaling pathway. TLR4, an innate immune receptor predominantly located on the surfaces of microglia and other cells, plays a pivotal role in identifying external and internal stimuli, thereby initiating inflammatory responses [38]. According to previous research, systemic and neurological inflammation in patients with ASD is brought on by elevated TLR4 expression in their B cells, over-activation of the NF-κB signaling pathway, and an increase in oxidative stress [39]. A recent study revealed unusual elevations in TLR4 and NF-κB in the intestinal tracts of BTBR mice, suggesting the TLR4 signaling pathway as a viable target for treating autism-related gastrointestinal dysfunctions [40]. Aligning with these discoveries, our study established that miR-137 diminished NF-κB levels in microglial cells exposed to LPS.

MSCs are a type of adult stem cells, and they have become powerful tools to treat diseases such as inflammation, tissue damage, and regeneration and post-injury repair [41]. MSC-EVs have a lower risk of immune rejection and tumor formation compared to MSCs, which greatly enhances their use in clinical disease treatment [42]. Based on previous studies, MSC-EVs not only have the ability to penetrate the blood–brain barrier due to their unique nanoscale and lipid membrane encapsulation advantages but also carry the immune and neuroprotective factors that are enriched in MSCs.

MSC-EVs have been found to contain 4850 gene products and 4150 miRNAs through mass spectrometry and microarray analysis [43]. A notable finding of the present study was that the treatment of the BTBR mouse model with MSC-EVs alleviated neuroinflammation and autism-like behaviors. Previous studies have shown that intranasal administration of MSC-EVs can improve autism-like behaviors in ASD mice model [44, 45], but the mechanism remains unclear. Our study identified the miR-146 within MSC-EVs could target to TRAF6 to alleviate phenotypes in BTBR mice. Likewise, miRNA-146 administered from MSC-EVs has been found to inhibit microglial neuroinflammation by inhibiting TRAF6 and IL-1 receptor-related kinase 1 in microglia in patients with Alzheimer’s disease [46]. Therefore, the results of the current study add to the evidence that miRNA-146a-5p in MSC-EVs is involved in the regulation of the neuroinflammation associated with ASD in addition to other types of disease.

In this study, EVs were administered intranasally to mouse brains. Intranasal administration has been viewed as a possible alternative technique to improve drug delivery to the CNS due to increased research on the development of drug delivery systems. Anatomically, the nasal cavity has a direct path to the brain, and after nasal administration, the drug can enter the cerebrospinal fluid without causing harm and exert its therapeutic effects there, avoiding the BBB by passing through the olfactory mucosa and entering the CNS, cerebrospinal fluid, or brain tissue. Nasal drug delivery is a current research hotspot for CNS drug delivery. Nasal drug delivery for CNS diseases also has significant advantages over traditional drug delivery methods such as oral administration and injection, including high drug bioavailability, noninvasiveness, high blood flow, large surface area available for drug absorption, ease of application, rapid onset of action, and avoidance of drug damage to the liver and gastrointestinal tract and first-pass effect in the liver [47, 48]. Currently, permeation and absorption enhancers, enzyme inhibitors, mucoadhesive, and hydrogel systems have been incorporated into nasal formulation to improve drug absorption and permeability and to increase the residence time in the nasal mucosa.

Although significant progress has been made in EV-based drug delivery systems, it should be noted that there are many challenges in optimizing the techniques to isolate highly purified EVs. To extract EVs on a large scale for subsequent functional verification, this study used the ultracentrifugation method to prepare EVs. However, this method may lead to EVs aggregation and damage their membrane structures and may alter their targeting and therapeutic effects. In addition, it is worth noting that to achieve the targeting ability of EVs, ligands are attached to their surfaces through chemical conjugation. These active targeting molecular combinational techniques and systems need to be evaluated for safety and efficacy. The present study demonstrated that MSC-EVs enhanced the efficiency of miR-137 delivery, suggesting that EV-based miR-137 delivery is a promising therapeutic strategy for the treatment of ASD.

## Conclusion

As a reliable carrier, MSC-EVs can encapsulate miR-137, a small molecule that can be introduced into BTBR mice by intranasal administration or co-cultured with microglia in vitro, thereby alleviating autism-like behaviors and inhibiting neuroinflammatory responses in the brain and microglia through the TLR4/NF-κB pathway in mice. Meanwhile, the MSC-EVs contained abundant miR-146a-5p, which targeted the TRAF6/NF-κB signaling pathway. Taken together, our study suggested that combining MSC-EVs with miR-137 holds promise for the therapy of ASD and may have potential for clinical application.

### Electronic supplementary material

Below is the link to the electronic supplementary material.

Supplementary Material 1: Figure S1: Immunofluorescence of Iba-1 expression in cerebellum of C57BL/6J and BTBR mice. Figure S2: The vector map of miR-137 overexpressed lentivirus.

Supplementary Material 2: Table S1. Overlap of GSE62594 upregulates differential genes and miR-137 target gene. Table S2. The primer sequences used in this study.

## Data Availability

The data and materials used during the current study are available from the corresponding author on reasonable request.
