# Supplementary material for: Synergistic effect of mesenchymal stem cell-derived extracellular vesicle and miR-137 alleviates autism-like behaviors by modulating the NF-κB pathway
Source: J Transl Med. 2024 May 13;22:446. doi: 10.1186/s12967-024-05257-w (PMC11089771; doi:10.1186/s12967-024-05257-w)
Supplement: Supplementary file 1 — Supplementary Material 1: Figure S1: Immunofluorescence of Iba-1 expression in cerebellum of C57BL/6J and BTBR mice. Figure S2: The vector map of miR-137 overexpressed lentivirus. [file 12967_2024_5257_MOESM1_ESM.docx]

Supplementary Information

**Synergistic effect of mesenchymal stem cell-derived extracellular vesicle and miR-137 alleviates autism-like behaviors by modulating the NF-κB pathway**

*Qian Qin^#^, Zhiyan Shan^#^, Lei Xing, Yutong Jiang, Mengyue Li, Linlin Fan, Xin Zeng, Xinrui Ma, Danyang Zheng, Han Wang, Hui Wang, Hao Liu, Shengjun Liang, Lijie Wu*, Shuang Liang**

**Supplementary Figure S1**


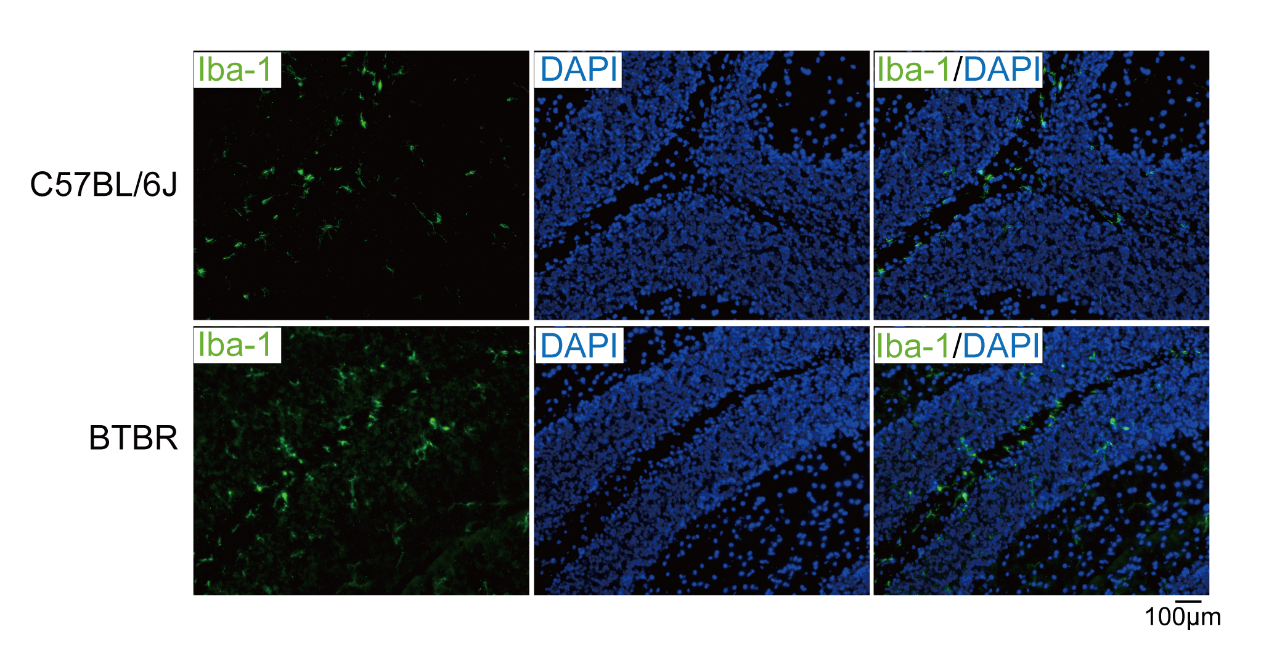


**Figure S1. Immunofluorescence of Iba-1 expression in cerebellum of C57BL/6J and BTBR mice.**

Scale bar: 100 μm.

**Supplementary Figure S2**


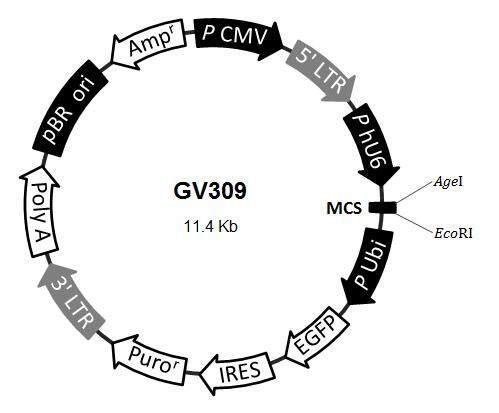


**Figure S2. The vector map of miR-137 overexpressed lentivirus.**
