# Supplementary material for: Synergistic effect of mesenchymal stem cell-derived extracellular vesicle and miR-137 alleviates autism-like behaviors by modulating the NF-κB pathway
Source: J Transl Med. 2024 May 13;22:446. doi: 10.1186/s12967-024-05257-w (PMC11089771; doi:10.1186/s12967-024-05257-w)
Supplement: Supplementary file 2 — Supplementary Material 2: Table S1. Overlap of GSE62594 upregulates differential genes and miR-137 target gene. Table S2. The primer sequences used in this study. [file 12967_2024_5257_MOESM2_ESM.docx]

Supplementary Information

**Synergistic effect of mesenchymal stem cell-derived extracellular vesicle and miR-137 alleviates autism-like behaviors by modulating the NF-κB pathway**

*Qian Qin^#^, Zhiyan Shan^#^, Lei Xing, Yutong Jiang, Mengyue Li, Linlin Fan, Xin Zeng, Xinrui Ma, Danyang Zheng, Han Wang, Hui Wang, Hao Liu, Shengjun Liang, Lijie Wu*, Shuang Liang**

**Table S1: Overlap of GSE62594 upregulates differential genes and miR-137 target gene.**

| **Gene symbol** | **T-statistical** | **P-value** | **FC** | **FDR** |
| --- | --- | --- | --- | --- |
| LATS1 | 20.7533 | 2.18E-08 | 8.73839 | 1.29E-05 |
| APLS3 | 5.861355 | 0.000314 | 3.821069 | 0.013046 |
| CLASP2 | 11.98654 | 8.18E-07 | 2.686023 | 0.000181 |
| KLF4 | 5.243474 | 0.000241 | 2.054808 | 0.010882 |
| FASL | 5.425177 | 0.000338 | 1.885897 | 0.013675 |
| SORL1 | 4.632162 | 0.000549 | 1.83932 | 0.019601 |
| **TLR4** | **5.148353** | **0.000196** | **1.787136** | **0.009424** |
| KLF15 | 4.953075 | 0.000417 | 1.63322 | 0.016198 |
| KIRREL | 4.278772 | 0.001985 | 1.568496 | 0.048693 |
| IQGAP1 | 4.898984 | 0.000632 | 1.522196 | 0.021414 |

**Table S2. The primer sequences used in this study.**

| Primer name | Primer sequence |
| --- | --- |
| GAPDH-Forward primer | CGTCCCGTAGACAAAATGGT |
| GAPDH-Reverse primer | TCAATGAAGGGGTCGTTGAT |
| Universal U6-Forward primer | CTCGCTTCGGCAGCACA |
| Universal U6-Reverse primer | AACGCTTCACGAATTTGCGT |
| has-miR-21-5p-Forward primer | ACACTCCAGCTGGGTAGCTTATCAGACTGA |
| has-miR-21-5p-Reverse primer | CTCAACTGGTGTCGTGGAGTCGGCAATTCAGTTGAGTCAACATC |
| has-miR-100-5p-Forward primer | ACACTCCAGCTGGGAACCCGTAGATCCGAA |
| has-miR-100-5p-Reverse primer | CTCAACTGGTGTCGTGGAGTCGGCAATTCAGTTGAGCACAAGTT |
| has-let-7f-5p-Forward primer | ACACTCCAGCTGGGTGAGGTAGTAGATTGT |
| has-let-7f-5p-Reverse primer | CTCAACTGGTGTCGTGGAGTCGGCAATTCAGTTGAGAACTATAC |
| has-let-7a-5p-Forward primer | ACACTCCAGCTGGGTGAGGTAGTAGGTTGT |
| has-let-7a-5p-Reverse primer | CTCAACTGGTGTCGTGGAGTCGGCAATTCAGTTGAGAACTATAC |
| has-miR-146a-5p-Forward primer | ACACTCCAGCTGGGTGAGAACTGAATTCCA |
| has-miR-146a-5p-Reverse primer | CTCAACTGGTGTCGTGGAGTCGGCAATTCAGTTGAGAACCCATG |
| miR137-3p-Forward primer | ACACTCCAGCTGGGTTATTGCTTAAGAATAC |
| miR137-3p-Reverse primer | CTCAACTGGTGTCGTGGAGTCGGCAATTCAGTTGAGCTACGCGT |
| Unified reverse primer | TGGTGTCGTGGAGTCG |
| KLF4-Forward primer | CCGACTAACCGTTGGCGT |
| KLF4- Reverse primer | GGTGGGTTAGCGAGTTGGAA |
| FASL-Forward primer | GAACCCCCACTCAAGGTCCAT |
| FASL-Reverse primer | TCAACCTCTTCTCCTCCATTAGC |
| IQGAP1-Forward primer | CTCGGCGCTGAACTCTAAGG |
| IQGAP1-Reverse primer | TTCAATCTCCAGAAGGACGCC |
| TLR4-Forward primer | CCATGCATTTGGCCTTAGCC |
| TLR4-Reverse primer | TGCAGCAGTCTACTGTGTGG |
| IL-1β-Forward primer | GTGTCTTTCCCGTGGACCTT |
| IL-1β-Reverse primer | CATCTCGGAGCCTGTAGTGC |
| IL-6-Forward primer | CTGCAAGAGACTTCCATCCAG |
| IL-6-Reverse primer | AGTGGTATAGACAGGTCTGTTGG |
| TNF-α-Forward primer | CCCTCACACTCACAAACCAC |
| TNF-α-Reverse primer | ATAGCAAATCGGCTGACGGT |
| IFN-γ-Forward primer | AGACAATCAGGCCATCAGCAA |
| IFN-γ- Reverse primer | CTCATTGAATGCTTGGCGCT |
